# The therapeutic effects of microRNAs in preclinical studies of acute kidney injury: a systematic review protocol

**DOI:** 10.1186/s13643-019-1150-1

**Published:** 2019-10-10

**Authors:** Sarah Zankar, Rosendo A. Rodriguez, Jose Luis Vinas, Kevin D. Burns

**Affiliations:** 1 Department of Medicine, The Ottawa Hospital and University of Ottawa, 501 Smyth Road, Ottawa, Ontario K1H 8L6 Canada; 2 Division of Nephrology, Department of Medicine, Kidney Research Centre, Ottawa Hospital Research Institute, University of Ottawa, 1967 Riverside Drive, Rm. 535, Ottawa, Ontario K1H 7W9 Canada

**Keywords:** Acute kidney injury, Animal models, microRNA, Preclinical, Ischemia, Exosomes, Extracellular vesicles, Therapy

## Abstract

**Background:**

Acute kidney injury (AKI) causes significant morbidity and mortality in humans, and there are currently no effective treatments to enhance renal recovery. MicroRNAs (miRNAs) are short chain nucleotides that regulate protein expression and have been implicated in the pathogenesis of AKI. Recently, preclinical studies in vivo have uncovered a therapeutic role for administration of specific miRNAs in AKI. However, the overall benefits of this strategy in preclinical studies have not been systematically reviewed, and the potential for translation to human studies is unclear.

**Aim:**

The primary aim is to conduct a systematic review of the therapeutic properties of miRNAs in preclinical studies of AKI. The secondary aim is to determine potential adverse effects of miRNA administration in these studies.

**Methods:**

A comprehensive search strategy will identify relevant studies in AKI in vivo models, using the MEDLINE, EMBASE, OVID, PUBMED, and Web of Science databases. The search strategy will include terms for mammalian (non-human) AKI models, including injury related to ischemia/reperfusion, nephrotoxicity, sepsis, contrast agents, cardio-pulmonary bypass, and hemorrhagic shock. Interventions will be defined as direct administration of exogenous miRNAs or antagonists of miRNAs, as well as maneuvers that alter expression of miRNAs that are mechanistically linked to AKI outcomes. The primary outcomes will be indices of kidney function and structure, and there will be no restriction on comparator interventions. Two independent investigators will initially screen abstracts, and selected articles that meet eligibility criteria will be reviewed for data abstraction and analysis. The SYRCLE RoB tool for animal studies will determine risk of bias, and meta-analysis will be performed as appropriate. The GRADE methodology will assess the quality of evidence.

**Discussion:**

The administration of selective miRNA mimics or antagonists exerts beneficial effects in mammalian models of AKI, although multiple obstacles must be addressed prior to translation to human clinical trials. The proposed systematic review will document key miRNA candidates, and determine effect size estimates and sources of outcome bias. The review will also identify gaps in knowledge and guide future directions in AKI research.

**Systematic review registration:**

PROSPERO CRD42019128854

## Background

The kidneys receive a high percentage of the cardiac output (25%) and are particularly sensitive to damage induced by sudden reductions in blood flow, as occurs with ischemia/reperfusion, sepsis or with exposure to nephrotoxins. Acute kidney injury (AKI) is a highly prevalent clinical disorder, characterized by rapid loss of kidney function occurring over hours to days, and is associated with significant morbidity and mortality [1]. Despite many decades of research, there are no established treatments that accelerate renal repair in humans [1]. Patients who recover from an episode of AKI are at risk for adverse long-term outcomes: a meta-analysis of 82 studies that included more than 2 million hospitalized adults followed for 1 year showed that patients with AKI have a threefold increased risk of new or progressive chronic kidney disease (CKD), a nearly fourfold increase in end-stage renal disease (ESRD) and almost twofold increase in mortality rates [2]. Indeed, the incremental health care utilization costs for AKI in Canada are estimated to be greater than $200 million per year [3]. Therefore, strategies to prevent AKI and facilitate kidney recovery are needed.

In recent years, preclinical studies have demonstrated beneficial effects of cell therapy (involving administration of progenitors or stem cells) in reducing tissue injury and accelerating repair pathways in AKI. Thus, delivery of endothelial progenitor cells (EPCs) or bone marrow-derived mesenchymal stromal cells (MSCs) has been shown to protect against AKI in animal models [4–7]. Despite these therapeutic effects however, engraftment of cells within injured tissues has not been consistently demonstrated, and protective effects have been attributed to paracrine mechanisms [7].

In this regard, extracellular microvesicles are leading candidates mediating the protective effects of cell therapy in models of ischemic kidney injury. Cantaluppi and colleagues showed that infusion of microvesicles derived from human EPCs protected rats from ischemic AKI and also prevented long-term development of CKD [8]. Furthermore, preclinical studies indicate that the use of MSC-derived microvesicles is strongly associated with improved organ function following injury. A systematic review of 6 preclinical studies showed that administration of human MSC-derived microvesicles (40–1000-nm diameter) in mouse and rat models of AKI improved blood urea nitrogen (BUN) and serum creatinine levels, increased kidney cell proliferation, and reduced tubular necrosis and cast formation [9].

**Fig. 1:**
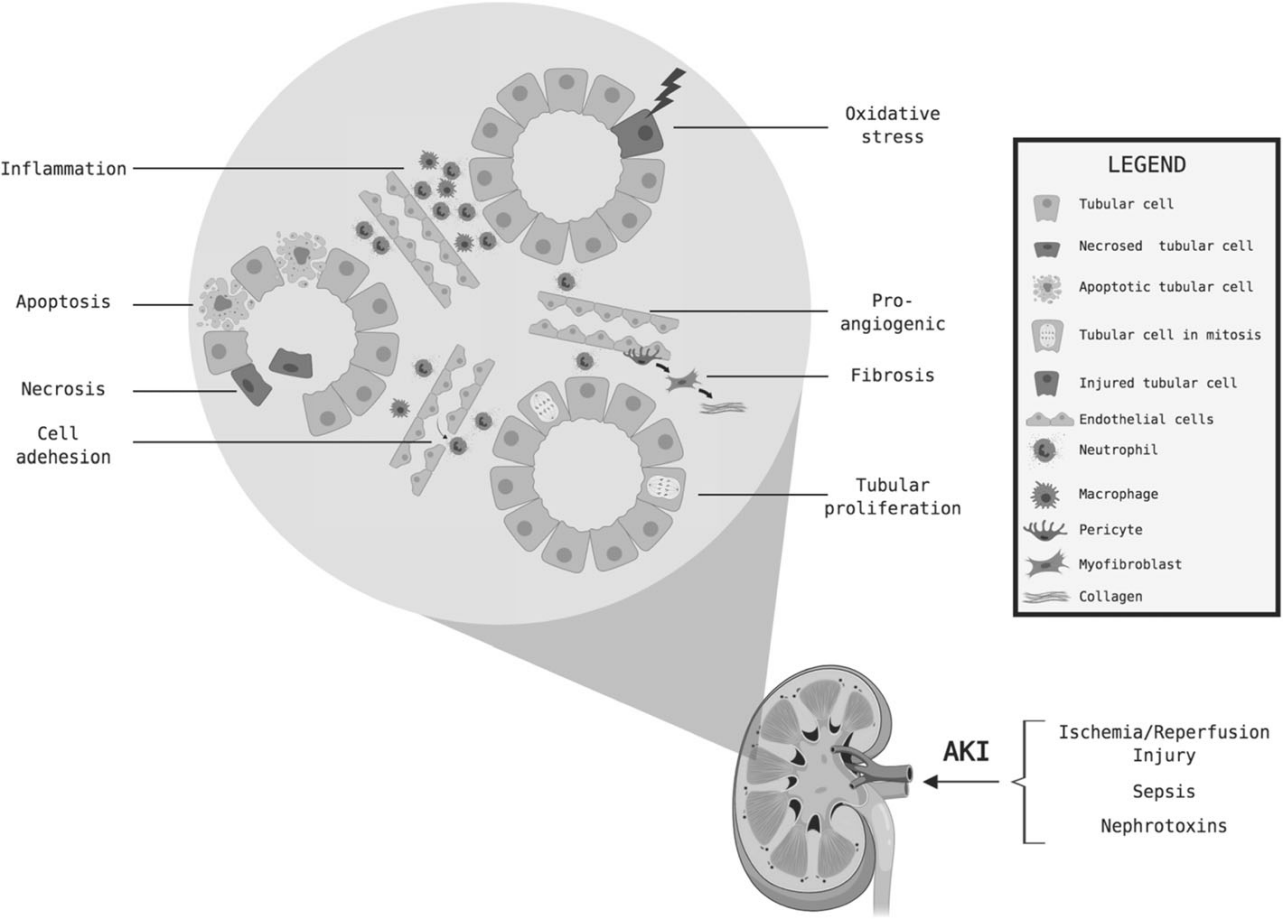
Pathways for miRNA involvement in acute kidney injury (AKI). Figure depicts schematic of pathways involved in the pathogenesis of AKI, highlighting kidney endothelial and tubular cell injury/death. MicroRNAs have been implicated in targeting messenger RNAs involved in each of these protective or pathogenic molecular pathways in experimental models of AKI

One distinct class of microvesicles is exosomes (diameter 40–100 nm), which are generated in the multivesicular body and then released from the cell [10]. Exosomes are packaged with cargo, including microRNA (miRNA), and upon binding to other cells the exosomal contents may be transferred, thereby modulating cell function. Several studies have revealed an important role for direct administration of candidate miRNAs or exosomal transfer of miRNAs in improving outcomes in experimental AKI [8, 11–13]. For instance, a potential candidate miRNA for treatment of AKI is miRNA-21, which has been shown to protect against ischemia-reperfusion kidney injury and apoptosis in mice, using a strategy involving administration of locked nucleic acid (LNA) antagonist of miRNA-21 [13]. Indeed, multiple miRNA species have been implicated in either protecting against AKI, or exacerbating renal injury, by targeting molecular pathways involved in angiogenesis, cell adhesion, apoptosis, inflammatory cell recruitment, and tubular cell proliferation [11] (Fig. 1).

Despite the growing evidence in preclinical studies for the therapeutic properties of miRNAs in AKI, no clinical studies involving miRNA therapeutics have been conducted in human AKI, and no trials are currently listed within the database of private and publicly funded studies worldwide (clinical.trials.gov). Barriers to translation of animal studies to clinical application may relate to methodological heterogeneity amongst preclinical studies, differences in therapeutic targets, variability in outcome measures, and relatively small sample sizes that prevent accurate estimation of effect size. A comprehensive systematic review of preclinical studies focused on the therapeutic effects of delivery of miRNAs or exosomal transfer of miRNAs in AKI would therefore facilitate estimation of overall efficacy and safety of these agents in animal models. Such a review would help identify sources of bias, reliable outcome measures, and study limitations that should be addressed prior to human clinical trials for AKI.

## Methods/design

This systematic review will be conducted in accordance with the Cochrane Collaboration Methods [14], Systematic Reviews Standards, and PRISMA guidelines [15]. The study protocol is registered in PROSPERO (www.crd.york.ac.uk/prospero) (registration number CRD42019128854).

### Aims

The primary aim of this systematic review is to determine the potential therapeutic properties of miRNAs in preclinical studies of AKI, with a focus on kidney functional and structural recovery/repair. The secondary aim is to determine potential adverse effects of miRNA administration in preclinical AKI studies.

### Design of systematic review

#### Animal species and models

The systematic review will include all mammalian species except humans, regardless of age or sex, with most studies likely to have been conducted in mouse or rat. Experimental models of AKI include (but are not limited to) ischemia/reperfusion injury, nephrotoxic injury (e.g., cisplatinum, warfarin, gentamicin, folic acid, glycerol-induced, aristolochic acid), and sepsis (lipopolysaccharide (LPS)-induced, cecal ligation and perforation), as well as contrast nephropathy [16], cardio-pulmonary bypass-induced AKI [17], and hemorrhagic shock [18]. Gene deletion, gene knockdown, or transgenic animal models will be included as appropriate. We will exclude models of chronic nephrotoxicity (e.g., cyclosporine-induced injury [19]) or renal fibrosis associated with the model of unilateral ureteral obstruction [20].

#### Interventions

The principal intervention will be defined as the direct exogenous administration of miRNAs, antagonists of miRNAs (antagomiRs) or locked nucleic acid (LNA) derivatives of miRNAs to the injured kidney, either as native nucleotides or as nucleotides within extracellular microvesicles or manufactured nanoparticles [21]. Administration will include intravascular (intravenous [i.v.] or intra-arterial [i.a.]), subcutaneous (s.c.), intraperitoneal (i.p.), intrarenal injections, or other routes. There will be no restrictions in dose and timing of administration.

Interventions that indirectly implicate miRNAs as potential therapeutic agents for AKI will also be reviewed. These will include in vivo interventions in the AKI model that alter the regulation/quantity of miRNAs, where these alterations are mechanistically linked to pathways or outcomes associated with AKI. We will exclude studies that are purely descriptive and that document changes in the pattern of miRNA expression levels associated with AKI, without an intervention to assess the impact of those changes on kidney function or structure.

#### Comparators

There will be no restriction on types of comparator interventions (e.g. placebo, sham, pharmacologic agents, scrambled miRNA, other).

#### Outcomes

The primary outcome will be kidney function, encompassing measures of estimated glomerular filtration rate (e.g., fluorescein isothiocyanate (FITC)-inulin clearance, serum creatinine), and BUN, as well as urinary and/or plasma markers of kidney injury, and kidney structural analyses by histologic assays (injury scores, inflammatory cell infiltration, apoptosis assays, immunohistochemistry). Changes in renal hemodynamic measures or vascular structure/function will be noted. Sex-specific outcomes will be recorded wherever possible. The secondary outcome will be measures of potentially adverse effects, including non-renal effects of the intervention, and mortality.

#### Languages

Articles written in English, French, Italian, or Spanish will be included.

#### Search strategy

We will follow the recommendations of the PRISMA statement [15] and the reporting guidelines provided by the Collaborative approach to meta-analysis and review of animal data from experimental studies (CAMARADES, www.camarades.info). A populated PRISMA-P checklist is provided in Additional file 1. The search strategy will identify studies in MEDLINE, EMBASE, OVID, PUBMED, and Web of Science databases, from 1946 to 2019. The reference list of included studies will be identified to look for additional study sources. We will exclude editorials, review articles, opinion papers, patent applications, and studies involving only in vitro experiments. The search strategy is depicted in Additional file 2 and will include MesH terms related to miRNAs, exosomes, extracellular vesicles, and AKI in experimental animal models.

#### Study screening

Titles and abstracts initially identified will be uploaded to Excel spreadsheets and screened for relevance independently by two investigators according to pre-defined criteria. Prior to formal screening, we will conduct a calibration exercise to optimize the screening questions. Eligibility will be determined by (a) clear identification of a therapeutic agent defined as direct exogenous administration in vivo of miRNAs, antagonists of miRNAs (antagomiRs), or LNA derivatives of miRNAs, either as native nucleotides or as nucleotides within extracellular microvesicles or manufactured nanoparticles; (b) indirect implication of miRNAs as potential therapeutic agents for AKI via in vivo interventions that alter the regulation or quantity of miRNAs, where these alterations are mechanistically linked to pathways or outcomes associated with AKI; (c) clear documentation of the animal model of kidney injury; and (d) use of functional and structural indices of kidney injury as outcome measures. Relevant articles will be retrieved for full-text assessment by two investigators, using specific inclusion and exclusion criteria (Table 1). To minimize discrepancies between reviewers, the exclusion criteria will be ranked from high to low priority and applied sequentially during the reviewing process with the highest excluding reason carefully documented. Any differences in classification between the two independent reviewers will be reviewed and a consensus decision made. In cases where consensus is not reached, a third author will be asked to provide an independent opinion, and a decision will be made based on this opinion. Duplicate citations will be eliminated manually by matching the title, authorship and citation information. In case of missing information about treatments, methodology, and/or outcomes of included studies, attempts will be made to contact the study authors by email or telephone.

**Table 1:** Inclusion and exclusion criteria for full-text analysis

Inclusion criteria	
a. Preclinical model of acute kidney injury b. In vivo mammalian model c. Direct administration of miRNAs, antagomiRs, or locked nucleic acids d. In vivo interventions that implicate miRNAs mechanistically in acute kidney injury e. Clearly identified functional and/or structural maker(s) of acute kidney injury obtained via validated methods and reported according to standard guidelines f. Any type of comparator g. Clear identification of the methods of analysis and time points of measurements	
Exclusion criteria in order of prioritization	
1. Not an animal study (e.g., in vitro models) 2. Not a model of experimental acute kidney injury 3. No administration of miRNA or derivatives or no mechanistic information related to miRNA (e.g., biomarker studies of miRNAs in AKI or descriptive studies on expression of miRNAs in AKI) 4. No outcome measures of kidney function or structure 5. Not original research (e.g., review paper, editorial, commentary, patent applications, letters to the editor, opinion papers, narrative reviews) 6. Not in English, French, Spanish, or Italian 7. Sub-studies of the main study	

#### Data abstraction

A data extraction form will be designed for gathering data and piloted between reviewers before its implementation. The data will be extracted manually from text, tables, and graphs onto the form by at least two of the authors and will include characteristics of the animal model (species, age, sex, AKI induction method, time points of analyses, number of animals), characteristics of the intervention and comparators (miRNA variant, dose, frequency, route of administration, carrier as specified), and outcomes (as described above). Timing and duration of administration of miRNAs represents a critical aspect to inform the design of future translational trials in humans. This is particularly relevant since in humans, the diagnosis of AKI often occurs after significant delay beyond the time of initial insult. Accordingly, we will also conduct sub-group analysis of data directed at the impact at time of administration of miRNA or variants, and/or duration of treatment. Subsequently, all collected data will be transferred manually into an Excel spreadsheet for data analysis.

#### Risk of bias and quality assessment

The SYRCLE RoB tool for animal studies will be used to assess risk of bias at the study and outcome levels [22], and key parameters that have been identified as threats to validity in preclinical studies will be assessed [23]. Study reporting bias will be assessed by 2 individuals, comparing outcomes reported in the methods and results sections of published reports, when full study protocols are not available.

#### Data synthesis and analysis

For data synthesis, we will provide a descriptive summary of all included studies. The variability on study design, methodological quality, type of therapeutic agent, AKI model, and statistical heterogeneity will be taken in consideration in our decision to perform a quantitative data synthesis on the primary outcome. If at least 2 studies report on the same primary outcome, a quantitative analysis (i.e., meta-analysis) will be performed on those studies. If the statistical heterogeneity between studies is high and/or its sources are not sufficiently explained, data will be reported descriptively. For each outcome measure (i.e., functional, structural) that is reported as continuous data, we will calculate the standardized or mean differences depending on individual study scales. The *I*^2^ statistic will be used to describe the percentage of variation across studies due to heterogeneity, rather than chance [24]. If the degree of heterogeneity between pooled studies is acceptable, weighted pooled effect estimates and their 95% confidence intervals will be calculated using the inverse variance statistical method and analyzed by the random effects model [25]. Depending on the number of eligible studies, we will compare the size of the effect estimates amongst different therapeutic agents (i.e., miRNAs, antagonists of miRNAs (antagomiRs), or LNA derivatives) by using the mean percent change (i.e., delta change) in the injury marker or the standardized mean differences and 95% confidence intervals between the intervention and comparator. If the delta change (%) is not provided, it will be calculated from the reported means and standard errors [26]. In cases of high statistical heterogeneity, we will attempt to identify sources of heterogeneity between studies using stratified and sub-group analyses. The significance of these sub-group differences will be determined by the Cochrane *X*^2^ test (*p* < 0.10). Data analyses will be performed using RevMan 5.3 (Copenhagen; The Nordic Cochran Centre, The Cochrane Collaboration; 2014).

#### Sensitivity, sub-group analyses, and publication bias

To reinforce the strength of our reviewing decisions, we are proposing sensitivity analyses according to the analysis method, outcomes, dose range, and time points of measurement. If during the process of the systematic review, we identify additional decision nodes, further sensitivity analyses will be performed. We are planning sub-group analyses to identify differences in the effect estimates according to the animal species, sex, model of kidney injury, and route of administration for the therapeutic agent. These analyses would facilitate our interpretation of the differences in effect size between studies and will help to identify the optimal preclinical model and delivery system. Reporting bias will also be assessed by funnel plots when at least 10 studies are available according to the outcome and intervention. Scatterplots of individual effect estimates against a measure of their precision will be displayed.

#### Confidence in cumulative estimates

If we find sufficient information in the literature, the GRADE methodology will be applied to assess the quality of evidence for at least 2 of the most important outcome measures. The recommendations will be classified as high, moderate, low, or very low. Limitations in current studies will be identified and suggestions for improvement provided.

If amendments to the study protocol are required, the date of each amendment will be recorded, followed by a description of the change as well as the rationale.

## Discussion

AKI has a relatively high prevalence and is associated with significant morbidity and mortality [27]. Despite recent advances in understanding the pathogenesis of AKI, there remains an urgent need for effective therapeutic strategies. Although miRNAs have been shown to play promising roles as biomarkers of disease severity in AKI, recent studies are also exploring their potential as therapeutic agents [28]. MiRNAs are highly homologous across multiple animal species, supporting their importance in regulating biological pathways [29]. Indeed, experimental studies reveal that some miRNAs act pathogenically and promote renal inflammation, apoptosis, and fibrosis in AKI, while others display anti-inflammatory, anti-apoptotic, and even pro-angiogenic effects, thereby protecting against renal injury [28]. While administration of specific miRNA mimics or antagonists has been shown to ameliorate or stabilize AKI in preclinical studies, a review of the therapeutic potential of miRNA therapy in AKI, and an assessment of the obstacles which need to be addressed before initiating clinical trials in humans, has yet to be conducted.

In progressing from preclinical studies to human therapies, several considerations must be addressed. For instance, animal models of AKI may not replicate the clinical features typically observed in humans with AKI. Thus, in preclinical studies, AKI is often induced by targeting of specific pathways via ischemia-reperfusion injury or administration of nephrotoxic agents, while in humans the causes of AKI are usually multifactorial [30]. Furthermore, human AKI often presents with other co-morbidities, such as CKD, diabetes, or heart failure [28], and there may be significant variability in responses and outcomes to similar insults. Predicting clinical outcomes in patients presenting with AKI is therefore difficult [28], and this is complicated further by genetic heterogeneity and differences in responses according to sex [30].

This systematic review represents an important first step in addressing the gap between preclinical studies and AKI in humans, and has the potential to uncover opportunities for first-in-human trials with miRNAs as therapeutic agents. Preclinical reports on AKI will be classified and compared for methodological heterogeneity, influence of animal sex, differences in therapeutic intervention and means of delivery of miRNAs, dosage, and time intervals for administration, outcome measures, and documented adverse events. By pooling results from multiple studies or interventions, effect size estimates can be documented, which will allow for more precise prediction of the utility of miRNA therapy in these models. In this regard, discordance between preclinical effect sizes and effect size in clinical trials is common. Practices such as unconcealed treatment allocation and unmasked outcome assessment may create a biased larger preclinical effect size [23], and therefore the systematic review will also include an assessment of sources of bias. Where sufficient data are available, a meta-analysis will be conducted to confirm the relationship between a specific miRNA and outcomes in animal models of AKI.

This systematic review has certain predictable limitations. Application of the risk of bias tool may uncover relatively few basic science studies that are sufficiently free of bias to allow for meta-analysis. Conducting the review will nonetheless be important to determine the current state of the field and identify study limitations associated with use of animal models of AKI. The review will also highlight the importance of rigorous efforts to limit bias or lack of transparency in data reporting for future preclinical research. Furthermore, significant study heterogeneity may exist, particularly in the specific miRNA(s) selected for interventional study in each manuscript. While several candidate miRNAs implicated in the pathogenesis of AKI have already been identified, it is important to recognize that individual miRNAs may target multiple messenger RNAs, and there may be homogeneity in pathways that are affected by distinct miRNA species. Thus, there is potential value in the proposed systematic review in attempting to identify common pathogenic mechanisms of action.

Finally, we acknowledge the limited focus of our review on preclinical data in AKI, without analysis of clinical observational studies involving measures of miRNAs in blood, urine, or kidney in humans with AKI. We consider that inclusion of human descriptive data in this review would create an abundance of information that would be difficult to separate, integrate, and analyze. Rather, upon completion of the systematic review of preclinical studies, we propose to conduct a separate scoping review, with a different methodological approach. This review will involve knowledge synthesis aimed at mapping key concepts relevant to the clinical utility of miRNA delivery in humans with AKI, guided by our findings from the preclinical literature. The scoping review will summarize the current literature with regards to miRNA measures in human AKI, examine the potential for conduct of a formal systematic review, identify gaps in knowledge, and make recommendations for future research directions. Building on the review of preclinical studies, such a qualitative review could inform optimal timing and duration of administration of miRNAs to humans with AKI, and identify most promising miRNA species for clinical trials.

## Supplementary information

**Additional file 1.** PRISMA-P 2015 Checklist

**Additional file 2.** Search strategy

## Data Availability

Not applicable

## Abbreviations

AKI: Acute kidney injury
BUN: Blood urea nitrogen
CKD: Chronic kidney disease
EPC: Endothelial progenitor cell
ESRD: End-stage renal disease
FITC: Fluorescein isothiocyanate
i.a.: Intra-arterial
i.p.: Intraperitoneal
i.v.: Intravenous
LNA: Locked nucleic acid
LPS: Lipopolysaccharide
miRNA: MicroRNA
MSC: Mesenchymal stromal cell
s.c.: Subcutaneous
