# Supplementary material for: The therapeutic effects of microRNAs in preclinical studies of acute kidney injury: a systematic review protocol
Source: Syst Rev. 2019 Oct 10;8:235. doi: 10.1186/s13643-019-1150-1 (PMC6788089; doi:10.1186/s13643-019-1150-1)
Supplement: Supplementary file 2 — Additional file 2. Search strategy [file 13643_2019_1150_MOESM2_ESM.docx]

**ADDITIONAL FILE 2**

Search strategy. <1946 to December 31, 2018.

Database: Embase Classic+Embase <1947 to 2018 January 02>, Ovid MEDLINE(R) ALL <1946 to December 31, 2018>

-------------------------------------------------------------------------------

1 exp MicroRNAs/ (186072)

2 (MicroRNA* or Micro RNA* or non coding rna* or noncoding rna* or Small Temporal rna* or miRNA* or mir).tw,kw. (245314)

3 mir.kf. (11880)

4 (microparticle* or ectosome* or exosome* or microvesicle* or extracellular vesicle*).tw,kw. (68208)

5 cell-derived microparticles/ or exosomes/ (26167)

6 or/1-5 (320418)

7 exp Acute Kidney Injury/ (118688)

8 (acute adj2 (renal or kidney)).tw. (115644)

9 ((acute or ischem* or ischaem* or reperfusion) and (kidney or renal)).kf. (8221)

10 ((ischem* or ischaem* or reperfusion) adj3 (renal or kidney)).tw. (21732)

11 aki.tw,kw. (26688)

12 ((acute or toxic or contrast induced) adj2 nephropath*).tw. (6320)

13 ((acute or toxic or contrast induced) and nephropath*).kf. (520)

14 nephrotoxi*.tw,kw. (53599)

15 or/7-14 (225622)

16 6 and 15 (1243)

17 limit 16 to yr="1990 -Current" (1236)

18 17 use medall (465)

19 exp microRNA/ (186072)

20 (MicroRNA* or Micro RNA* or non coding rna* or Small Temporal rna* or miRNA* or mir).tw. (228445)

21 exosome/ (17725)

22 membrane microparticle/ (4096)

23 (microparticle* or ectosome* or exosome* or microvesicle* or extracellular vesicle*).tw. (66231)

24 19 or 20 or 21 or 22 or 23 (303849)

25 exp Acute Kidney Injury/ (118688)

26 (acute adj2 (renal or kidney)).tw. (115644)

27 aki.tw. (26352)

28 kidney ischemia/ (10463)

29 contrast induced nephropathy/ (4241)

30 ((acute or toxic or contrast induced) adj2 nephropath*).tw. (6320)

31 ((ischem* or ischaem* or reperfusion) adj3 (renal or kidney)).tw. (21732)

32 nephrotoxicity/ (60934)

33 or/25-32 (238340)

34 24 and 33 (1161)

35 limit 34 to yr="1990 -Current" (1156)

36 35 use emczd (798)

37 18 or 36 (1263)

38 remove duplicates from 37 (882)

39 38 use medall (461) Medline

40 38 use emczd (421) Embase

Web of Science – up to January 3^rd^, 2019
